# Radiation Therapy in Extensive Stage Small Cell Lung Cancer

**DOI:** 10.3389/fonc.2017.00169

**Published:** 2017-08-11

**Authors:** Branislav Jeremic, Antonio Gomez-Caamano, Pavol Dubinsky, Nikola Cihoric, Franesc Casas, Nenad Filipovic

**Affiliations:** ^1^BioIRC Centre for Biomedical Research, BioIRC, Kragujevac, Serbia; ^2^Hospital Clínico Universitario, Complejo Hospitalario Universitario de Santiago, Santiago de Compostela, Spain; ^3^East Slovakia Institute of Oncology, Louis Pasteur University Hospital, Kosice, Slovakia; ^4^Department of Radiation Oncology, Inselspital, Bern University Hospital, University of Bern, Bern, Switzerland; ^5^Hospital Clínic de Barcelona, Barcelona, Spain

**Keywords:** extensive disease, small cell lung cancer, thoracic radiotherapy, chemotherapy, prophylactic cranial irradiation

## Abstract

Lung cancer is the major cancer killer in the Western world, with the small cell lung cancer (SCLC) representing around 15–20% of all lung cancers. Extensive disease small cell lung cancer (ED SCLC) is found in approximately two-thirds of all cases, composed of both metastatic (M1) and non-metastatic (but presumably with tumor burden too large for locoregional-only approach) variant. Standard treatment options involve chemotherapy (CHT) over the past several decades. Radiation therapy (RT) had mostly been used in palliation of locoregional and/or metastatic disease. In contrast to its established role in treating metastatic disease, thoracic RT (TRT) had never been established as important part of the treatment aspects in this setting. In the past two decades, thoracic oncologists have witnessed wide introduction of modern RT and CHT aspects in ED SCLC, which led to more frequent use of RT and rise in the number of clinical studies. Since the pivotal study of Jeremic et al., who were the first to show importance of TRT in ED SCLC, a number of single-institutional studies have reconfirmed this observation, while recent prospective randomized trials (CREST and RTOG 0937) brought more substance to this issue. Similarly, the issue of prophylactic cranial irradiation was investigated in EORTC and the Japanese study, respectively, bringing somewhat conflicting results and calling for additional research in this setting. Future studies in ED SCLC could incorporate questions of RT dose and fractionation as well as the number of CHT cycles and type of combined Rt-CHT (sequential vs concurrent).

## Introduction

Lung cancer is the major cancer killer in the Western world (1), with the small cell lung cancer (SCLC) representing around 15–20% of all lung cancers (2). While its incidence is declining in men, it continues to rise in women (3). In spite of significant efforts and refinements in staging system (4), division by “extensiveness” of the disease is still widely used. Extensive disease small cell lung cancer (ED SCLC) is found in approximately two-thirds of all cases, composed of both metastatic (M1) and non-metastatic (but presumably with tumor burden too large for locoregional-only approach) variant. Standard treatment options involve chemotherapy (CHT) over the past several decades (5). Various efforts to optimize treatment outcome with CHT, such as maintenance CHT or higher CHT doses unequivocally failed (6–10). With CHT, the median survival times (MST) are 9–12 months, while 5-year survivals of only 1–2% (9, 11–13).

Radiation therapy (RT) had mostly been used in palliation of locoregional and/or metastatic disease. In contrast to its established role in treating metastatic disease, thoracic RT (TRT) had never been established as important part of the treatment aspects in this setting. This was largely due to conflicting reports in the past several decades about its usefulness in controlling intrathoracic tumor burden (14–17), and predominantly metastatic nature of the disease. It is likely that inferior diagnostic and staging tool as well as outdated RT and CHT aspects such as 2D RT planning, modest RT doses, and non-platinum-based CHT significantly contributed to poor RT performance in this setting.

## Thoracic RT

In the past two decades, thoracic oncologists have witnessed wide introduction of modern RT (3D planning, altered RT fractionation) and CHT (platinum-based) aspects in ED SCLC. This has resulted in more frequent use of RT and, importantly, rise in the number of clinical studies addressing the issue of optimization of treatment approaches by focusing on important aspects of RT.

The turning point in the history of modern treatment of ED SCLC occurred with the publication of seminal paper of Jeremic et al. (13) in 1999. It was the very first study which tested, in a prospective randomized Phase III fashion, standard treatment option (CHT) vs CHT and TRT, with a prophylactic cranial irradiation (PCI) given in both arms. This trial was based on observations from the past studies that, in ED SCLC, there were frequent intrathoracic failures, which also frequently occur even in patients who achieved initial complete response (CR) after CHT. Hence, study of Jeremic et al. (13) had its premises in the following: (1) significant proportion of patients with ED SCLC experience intrathoracic (locoregional) treatment failure, which cannot be successfully treated with second line CHT, (2) these failures may also become the source of subsequent metastatic disease (in patients with previous non-metastatic ED SCLC) and lead to death, (3) TRT could control intrathoracic tumor burden, (4) if successful, this may lead to improved and prolonged intrathoracic tumor control, and, if significant, may lead to an improvement in overall survival. Ultimate question, overshadowing all these considerations was: which subgroup of patients may have been suitable for testing the place and role of TRT in ED SCLC.

In the Jeremic trial (13), subjects were adult treatment-naive patients with good PS and biopsy-proven ED SCLC. All patients initially received three cycles of standard-dose cisplatin/etoposide (PE) regimen, after which complete patient reevaluation and restaging was performed both at local (intrathoracic) and the distant level. Randomization included only patients who achieved either a CR at both local and distant level, labeled as CR/CR or those who achieved a partial response (PR) within the thorax accompanied with the CR elsewhere (labeled as PR/CR). They received either accelerated hyperfractionated RT (Acc Hfx RT) and concurrent low-dose daily CHT, given on each RT day, followed by PCI, and then by additional two cycles of PE (group I) or four additional cycles of PE and PCI (Group II). Patients achieving worse response were not randomized. Total tumor dose was 54 Gy in 36 fractions, 1.5 Gy BID, while PCI dose was 25 Gy in 10 daily fractions. When deemed appropriate, palliative RT was given to patients with metastatic lesions with 30 Gy in 10 daily fractions.

A total of 210 patients entered this study. TRT added to CHT offered superior outcome over CHT alone in terms of both the MST and 5-year survival rates [17 vs 11 months (*p* = 0.041), and 9.1 and 3.7%, respectively]. Similar was observed for the local recurrence-free survival (the median time to local recurrence, 30 vs 22 months, and 5-year local recurrence-free survival, 20 vs 8.1%, respectively; *p* = 0.062), but RT added to CHT did not offer better distant metastasis-free survival (*p* = 0.35).

To enlighten the effects of TRT on local (intrathoracic) level, local CR rates were evaluated after three cycles of induction CHT at week 9 (i.e., just before the randomization), week 15 (i.e., when either Acc Hfx RT/CE in group I or 2 additional cycles of PE in group II were administered), and at week 21 (2 more cycles of CHT in each group). Local CR rate was similar after 9 weeks (47 vs 44%, *p* = 0.77), but after week 15, the local CR rate became significantly higher in group I than in group II (96 vs 61%, *p* = 0.000007). This was maintained at week 21 (96 and 66% for the two groups, respectively; *p* = 0.00005). Therefore, fourth and the fifth cycles of CHT barely improved response in group I once Acc Hfx RT/CE had been given. Similarly, the sixth and seventh cycles of PE in group II brought only a few percent increase in response rates. What these results imply is that perhaps one may not need more than 3–4 CHT cycles in this patient population, possible food for thoughts for future trials.

Jeremic et al. (13) were the first to show that TRT plays indispensible role in the treatment of patients with ED SCLC after initial CHT. Beside primary study endpoints, an analysis of various pretreatment prognostic factors showed that higher KPS score and no significant weight loss were strong prognosticators of improved treatment outcome. As a potential guide for future studies, the number of metastases independently influenced survival. It was shown that metastatic tumor burden should be taken into account since patients with ≥2 metastases had significantly worse outcome than those with only one metastasis. Since approximately 90% of all patients in the study of Jeremic et al. (13) had 1–2 metastases, subsequent discussions in this field frequently labeled this disease extent as *limited extensive disease*.

Overall impact of the study of Jeremic et al. (13) was not easy to comprehend in the years following its publication. However, 10 years after its publication, the study of Ou et al. (18) retrospectively analyzed the data from several counties in southern California with estimated population of 6.2 million. Of a total of 3,428 ED SCLC patients, RT was given to 1,204 (35.1%) patients. For this group, the 2-year, and MST were 9.3%, and 8 months, respectively, being significantly better than in those who did not receive RT (3.8%, and 4 months, respectively; *p* < 0.0001). Analysis of prognostic factors showed that delivered RT exerted independent and positive influence of on treatment outcome (*p* < 0.001).

A recent survey of 473 practicing US radiation oncologist attempted to identify the current pattern of practice of TRT in ED SCLC (19). In spite of great variation in the patient selection and doses of RT used, TRT was recommended after systemic CHT by 96% of the respondents. The type of the institution influenced the decision with patients treated in private clinics being more likely to receive TRT than patients treated at academic centers (*p* = 0.0101). Interestingly, lower TRT recommended doses were associated with respondents claiming higher self-rated knowledge of individual clinical trials.

Past several years also brought studies that investigated the same issue. In a prospective study from Canada (20), the median time to disease progression was 8.4 months and the MST was 13.7 months. Additionally, two single-institutional, retrospective studies showed the same. In the Chinese study (21), for TRT-treated group MST was 17.2 months, and 5-year survival was 10.1%, respectively (*p* = 0.0001), while another Canadian study (22) reported on MST of 14 months and 2-year survival of 14% for TRT/CHT treatment.

Recently, in an EORTC (23) prospective phase III study patients with World Health Organization PS of 0 to 2 and confirmed ED SCLC without clinical evidence of brain, leptomeningeal, or pleural metastases, who achieved any response to 4–6 cycles of PE were treated with either TRT (30 Gy in 10 fractions) or no TRT, while all patients receiving PCI. Overall survival was longer in the TRT arm (*p* = 0.066), whereas 12-, 18-, and 24-month survival rates in the 2 arms were 33, 16, and 13% vs 28, 16, and 3%, respectively. Although the trial was negative for the primary endpoint of 1-year overall survival, significance was achieved at 18 months (*p* = 0.03) and was maintained at 24 months (*p* = 0.004). MST from the time of randomization was 8 months, but when calculated from diagnosis, it was 12 months. Progression-free survival was longer in the TRT arm (*p* = 0.001). Intrathoracic progression (isolated or accompanied by progression elsewhere or as the first site of disease progression) was seen less frequently in the TRT arm. Almost a 50% reduction in intrathoracic recurrences (80 vs 44%, respectively; *p* = 0.001) was observed.

Although EORTC study (23) characteristics (study design, patient eligibility, treatments offered) differed from those of Jeremic et al. (13) (Table 1), it is tempting to discuss and compare the two studies (Table 2) in order to obtain better perspective for future studies planning and execution. EORTC study (23) reconfirmed the importance of local control as initially suggested by Jeremic et al. (13). However, more intensive TRT given with concurrent CHT in a shorter OTT in the study of Jeremic et al. (13) may have led to faster improvement in local control which, in turn, may have led to faster improvement in the overall survival. More intensive tumor cell kill was accompanied with somewhat higher incidence of acute toxicity, which in both studies was acceptable and, in the study of Jeremic et al. (13), only high-grade acute esophageal toxicity was significantly more frequent in the TRT group. What this attempted comparison hints at is the large gap in both the patients’ and the treatment aspects when considering the two studies’ characteristics (13, 23) perhaps being at the two extremes, hence the necessity to fill in the existing gap with more clinical research.

**Table 1:** Patient and treatment characteristics.

Issue	Jeremic et al.	CREST
Less favorable patients (PS2)	0%	10%
Initial (pre-randomization) CHT	3 cycles	6 cycles
TRT (dose/fx)	54 Gy/36fx BID—Hyperfx	30 Gy/10fx QD—Hypofx
CHT-TRT	CHT followed by concurrent TRT-CHT	CHT followed by TRT (no concurrent part)
PCI—TRT	PCI followed TRT-CHT	Concurrent in almost 90% pts

*CHT, chemotherapy; TRT, thoracic radiotherapy; fx, fraction; BID, hyperfractionation (2 fx a day); QD, conventional fractionation (1 fx a day); PCI, prophylactic cranial irradiation*.

**Table 2:** Outcomes.

Issue	Jeremic et al.	CREST	Comments
Importance of improved LC	Yes	Yes	Leads to improved OS
Tempo of achieving improvement of LC	Faster	Slower	Leads to a faster improvement of OS in Jeremic et al.
OTT	Shorter	Longer	Possible due to better patient characteristics in Jeremic et al.
Incidence of high-grade toxicity	5% (lung) 20% (esophagus)	1.2% (lung) 1.6% (esophagus)	Higher TRT doses and concurrent CHT in Jeremic et al.
Duration of CHT	Shorter	Longer	Shorter appropriate in favorable patients?

*LC, local control; OS, overall survival; OTT, overall treatment time; TRT, thoracic radiotherapy; CHT, chemotherapy*.

One such attempt have been materialized in the Radiation Therapy Oncology Group study 0937 (24) during which patients with 1–4 extracranial metastases were deemed eligible after achieving either CR or PR to initial CHT. Patients received either PCI alone or PCI + TRT to the thorax and metastases. PCI was given with 25 Gy in 10 daily fractions in 2 weeks, while TRT was given with 45 Gy in 15 daily fractions in 3 weeks. Between March 2010 and February 2015, a total of 86 patients were randomized. The study crossed the futility boundary for OS and was closed at planned interim analysis prior to meeting accrual target. With the median follow-up of 9 months, 1-year overall survival was similar between the groups: 60.1% (95% CI: 41.2–74.7%) for PCI and 50.8% (95% CI: 34.0–65.3%) for PCI + + TRT (*p* = 0.21). Three and 12-month rates of progression were 53.3 and 79.6% for PCI, and 14.5 and 75% for PCI + TRT. Time to progression favored PCI + TRT (*p* = 0.01). Not to be forgotten, there were some imbalances in the two groups, which might better explain the negative results for overall survival despite improvements in thoracic control, including the higher number of patients with PR vs CR to CHT and 2–4 vs 1 metastases in the group receiving RT. Treatment-related toxicity was also similar between the two arms. The authors concluded that overall survival exceeded predictions for both arms with the consolidative RT delaying progression but not improving the 1-year overall survival.

Although this trial will definitely be seen as a negative trial, it brought several important findings. The first site of failure after CHT is likely to be in sites of presenting disease; RT to these sites alters failure patterns; late RT without concurrent CHT is not durable; and, oligometastatic ED SCLC survival seems to again approach that of LD SCLC, confirming the postulates and results of Jeremic et al. (13). Ineffective RT dose and schedule, advanced age, and an imbalance in disease burden in the two groups all likely contributed to lack of survival advantage with consolidative RT in this trial. In addition, considering all trial aspects, authors suggested that perhaps a more appropriate treatment for this patient population with low volume systemic disease could have been early RT concurrent with cycle three or four of CHT in patients with a favorable response to cycles one and two of CHT followed by PCI, similar to the Jeremic trial (13).

## Prophylactic Cranial Irradiation

Contrary to the place and role of PCI in limited disease SCLC, where several PRCTs and MAs exist, in ED SCLC data concerning it is much more limited. A large PRCT of EORTC included responders to 4–6 cycles of CHT (25). In this trial, patients were randomized to receive either PCI (20 Gy in 5 daily fractions or 30 Gy in 12 daily fractions) or observation. PCI offered significantly lower cumulative risk of brain metastasis at 1-year (14.8 vs 40.4%, *p* < 0.001), which led to an improvement in the progression-free survival (14.7 vs 12 weeks, respectively, *p* = 0.02). Finally, PCI led to an improvement in 1-year overall survival (27 vs 13%, respectively, *p* = 0.003) as a consequence of improved CNS control.

The same EORTC trial (25, 26) collected self-reported patient data using both Quality of Life Questionnaire C30 and Quality of Life Questionnaire Brain Cancer Module while investigating the effect of PCI on quality of life (QoL). In the first report, side-effects of PCI, including fatigue and hair loss, were significantly more severe in the group of patients receiving PCI (25). However, no significant differences were seen in the remaining endpoints. Importantly, in a subsequent report, there was a limited effect of PCI on these factors, none reaching the level of clinical significance as (26). Severe worsening in global health status (35 vs 22%) from base line up to 3 months was observed in the PCI group; however, one must not forget that there was a 94% participation rate at baseline, followed by poor compliance during the follow-up (60 and 55% at 6 weeks and 3 months, respectively). However, the control arm was significantly superior when an exploratory analysis of other symptom scale factors was performed.

These results have profoundly influenced the practice of RT in ED SCLC as PCI was overwhelmingly accepted as standard of treatment in this setting (5, 27). However, fresh data from a Japanese trial (28) seem to question that, in that trial, patients with any response to platinum-based doublet CHT and no brain metastases on MRI received PCI (25 Gy in 10 fractions) or observation. All patients were required to have brain MRI at 3-month intervals up to 12 months and at 18 and 24 months after enrollment. The primary endpoint was overall survival. 224 patients were randomly assigned. In the planned interim analysis, of the first 163 enrolled patients, Bayesian predictive probability of PCI being superior to observation was 0.011%, resulted in early termination of the study because of futility. In the final analysis, the MST was 11.6 months in the PCI group and 13.7 months in the observation group (*p* = 0.094). The most frequent grade ≥3 adverse events at 3 months were anorexia, malaise, and muscle weakness in lower limbs, which were all similar between the two groups. No treatment-related deaths occurred in either group.

While the Japanese study (28) reconfirmed the importance of CNS metastasis control, it failed to observe its influence on overall survival. Except, perhaps, fewer patients in that study, other possible reasons may exist as explanations for the existing discrepancy between Japanese (28) and EORTC (25) study. Japanese patients with ED SCLC were enrolled after they had been confirmed not to have brain metastases by MRI before randomization. By contrast, in the EORTC study (25), brain imaging at diagnosis was available in only 29% of randomized patients, while the proportion of patients who had brain imaging just before randomization was not stated. It is also very likely that in the EORTC study (25), some randomized patients actually had asymptomatic brain metastases before randomization since mandatory staging and follow-up procedures did not include brain imaging unless suggestive clinical symptoms were present. The longer overall survival reported by the EORTC study (25) in the PCI group might have reflected responses of asymptomatic brain metastases that had already been present before randomization. Although observation group encountered higher incidence of brain metastases than the PCI group in the Japanese trial (28), this did not result in shorter survival in the observation group, which contrasts EORTC study (25) findings. Possible explanation for this difference may be in difference in the proportion of patients who received subsequent treatment. Eighty-eight percent of patients in the PCI group and 89% of patients in the observation group received second-line CHT, however, more patients in the observation group received third-line or fourth-line CHT than did those in the PCI group. Additionally, in the Japanese study (28), anorexia, nausea, and malaise, which could be caused by CHT, were frequent and severe in patients in the PCI group beyond 3 months after randomization. The persistence of these adverse events, and the resultant impairment in QoL during subsequent CHT, might have decreased the feasibility and tolerability of such treatment in the PCI group in that study. The PCI group and the observation group had similar overall survival probably because of this decreased feasibility and tolerability. Also, the higher frequency of brain metastases seen in Japanese study (28) is mainly attributable to the detection of asymptomatic brain metastases by MRI. Not to be forgotten, the difference in the proportions of patients who had subsequent therapy between the two studies is presumably because some patients with symptomatic brain metastases in the control group of the EORTC study (25) would not have had subsequent CNS RT or CHT because of deterioration in their general condition, whereas patients with asymptomatic brain metastases detected by MRI in the CREST study did receive both CNS RT and subsequent CHT. Finally, the patients in the PCI group had more liver metastases, likely negatively to influence overall survival. Considering the impact of two studies on daily clinical practice, one may perhaps conclude that the Japanese study (28) may now slightly erode the firm position PCI had had in the past several years since the publication of the EORTC study (25) as “non-believers” would now how have somewhat stronger rationale against the use of PCI. It is reasonable to expect that these results would call for additional studies with more uniform diagnostic and follow-up criteria, including precise documentation of QoL aspects, which must take into account therapy administered at the time of CNS progression.

## Future Tasks

Research interests in the field of ED SCLC seem to have been revived in the past decade, after a dry decade post-Jeremic trial (13). Both PRCTs and single-institutional studies clearly show that thoracic oncologists understood the implication of the Jeremic trial (13). Indeed, in spite of CREST (23) and RTOG0937 (24) controversies, more emphasis is and will be made on the place and role of RT in this setting, including employment of modern RT technologies (29). Future studies should address important RT-related (optimal TRT and extrathoracic RT dose/fractionation and its timing) and CHT-related questions (number of cycles and its concurrent vs sequential administration). They may include, but are not limited to the following: palliative vs curative TRT dose; sequential vs concurrent RT-CHT; concurrent RT-CHT at cycle 3 vs concurrent RT-CHT at cycle 5; total of 4 vs total of 6 cycles of CHT.

## Author Contributions

Study design: BJ. Drafting of manuscript: BJ, AG-C, PD, NC, FC, and NF. Major revision of the manuscript, and final critique of the manuscript: BJ, AG-C, PD, NC, FC, and NF. Finalization of manuscript: BJ.

## Conflict of Interest Statement

The authors declare that the research was conducted in the absence of any commercial or financial relationships that could be construed as a potential conflict of interest.
